# Romantic Attachment, Internalized Homonegativity, and Same-Sex Intimate Partner Violence Perpetration Among Lesbian Women in Italy

**DOI:** 10.3389/fpsyg.2022.870921

**Published:** 2022-04-07

**Authors:** Giacomo Tognasso, Tommaso Trombetta, Laura Gorla, Shulamit Ramon, Alessandra Santona, Luca Rollè

**Affiliations:** ^1^Department of Psychology, University of Milano-Bicocca, Milan, Italy; ^2^Department of Psychology, University of Torino, Turin, Italy; ^3^Department of Allied Health, Midwifery and Social Work, University of Hertfordshire, Hatfield, United Kingdom

**Keywords:** Same-Sex Intimate Partner Violence, perpetration, romantic attachment, internalized homonegativity, minority stress, LGBT+, lesbian women

## Abstract

Same-Sex Intimate Partner Violence (SSIPV) among lesbian women has been underestimated until few decades ago. While the association between romantic attachment and SSIPV has been widely demonstrated, mechanisms that mediate this association and the complex relationships between romantic attachment, SSIPV, and SSIPV-specific risk factors have not been adequately investigated to date. The current study assessed the influence of romantic attachment on SSIPV perpetration among lesbian women, exploring the mediating role of internalized homonegativity within this association. Three hundred and twenty-five Italian lesbian women with a mean age of 30 years were recruited and completed the following self-report measures: the Experiences in Close Relationships-Revised (ECR-R), the Measure of Internalized Sexual Stigma, and the Revised Conflict Tactics Scale Short Form. The results showed a positive association between attachment anxiety, and general and psychological SSIPV perpetration. Similarly, attachment avoidance was positively related with general, psychological, and physical SSIPV perpetration. The association between romantic attachment, and general and psychological SSIPV was partially mediated by internalized homonegativity. These findings have theoretical implications and provide valuable information to implement services and interventions tailored for SSIPV, to date scarce and not effective.

## Introduction

Gender-based violence or Violence Against Women (VAW) has been object of interest since the 1970s. Second-wave feminism increased attention toward this phenomenon, recognizing it as a complex social issue (McClennen, 2005; Rollè et al., 2021). In contrast, Intimate Partner Violence (IPV) among lesbian women, and more in general Same-Sex Intimate Partner Violence (SSIPV), have been largely underestimated until few decades ago (Edwards et al., 2015; Estes and Webber, 2017; Rollè et al., 2020). Despite the lack of attention by public opinion and researchers toward this phenomenon, in a representative study of the United States population by Walters et al. (2013), 36.3 and 63.0% of lesbian women reported to experience within a romantic relation physical and psychological abuse respectively. In addition, no differences emerged when IPV prevalence rate among lesbian women was compared with that among heterosexual women.

Although IPV entails negative consequences on the physical and psychological wellbeing of victims, only few studies explored risk factors associated with couple violence perpetration among same-sex couples. Within these, SSIPV was associated with psychological (e.g., depression, self-esteem, and romantic attachment), relational (e.g., dyadic adjustment, relationship satisfaction, and fusion), family of origin-related and sexual minority-specific factors (e.g., internalized homonegativity; LGB+ community support), substance use, and sexual behaviors (Balsam and Szymanski, 2005; Bartholomew et al., 2008; Stults et al., 2015; Gabbay and Lafontaine, 2017a; Stephenson and Finneran, 2017; Miltz et al., 2019; Wei et al., 2020; Sharma et al., 2021). Further shed light on risk factors of SSIPV perpetration can inform services and social policies in order to decrease its prevalence and relapses.

### Romantic Attachment and Same-Sex Intimate Partner Violence

Romantic attachment refers to the bond between intimate partner that promotes physical closeness and emotion regulation in time of fear, threat, and distress (Hazan and Shaver, 1987). Unlike childhood attachment from which it stems (which has the same function, but it is asymmetrical in nature), romantic attachment implies a symmetrical relation in which both partners rely on the other as a safe heaven and secure base (Santona et al., 2019). It is mainly assessed along two dimensions (Brennan et al., 1998; Fraley et al., 2000): attachment anxiety, which is characterized by fear of rejection, fear of abandonment, and separation anxiety, and it is associated with the hyperactivation of the attachment system; and attachment avoidance, which is concerned with discomfort with closeness and fear of intimacy, and it is related with the deactivation of the attachment system (Mikulincer and Shaver, 2005).

Romantic attachment has been found as a predictor of IPV perpetration among both heterosexual and sexual minorities (Bartholomew et al., 2008; Derlega et al., 2011; Gabbay and Lafontaine, 2017a,b; Velotti et al., 2018; Santona et al., 2019). From the attachment perspective, couple violence can be seen as an extreme attempt to regulate affect and physical distance from the partner. According with this assumption, people with high levels of attachment anxiety seem to perpetrate IPV as an attempt to maintain closeness with the partner (Allison et al., 2007; Bartholomew et al., 2008; Gabbay and Lafontaine, 2017a). In contrast, people with high levels of attachment avoidance can resort to IPV with the aim to avoid closeness and the fear of intimacy it entails (Allison et al., 2007; Gabbay and Lafontaine, 2017a).

### Romantic Attachment, Internalized Homonegativity, and Same-Sex Intimate Partner Violence

While the relation between romantic attachment and IPV is well-documented, little attention has been paid to mechanisms through which it influences the risk of SSIPV perpetration among lesbian women and to the complex relation between romantic attachment and other risk factors that characterize SSIPV. Within this class of variables (i.e., SSIPV-specific risk factors), internalized homonegativity, which refers to negative affect and attitudes directed toward the self and one’s personal sexual orientation (Meyer, 1995, 2003), seem to take a main role in predicting IPV perpetration among sexual minorities. Several studies identified a positive association between levels of internalized homonegativity and SSIPV perpetration (Balsam and Szymanski, 2005; Edwards and Sylaska, 2013; Suarez et al., 2018; Miltz et al., 2019). As reported by authors (Byrne, 1996; Cruz and Firestone, 1998; Bartholomew et al., 2008) and drawing from a psychodynamic conception of violence (Glasser, 1998; Fonagy, 1999; Asen and Fonagy, 2017; Yakeley, 2018), the internalization of the sexual stigma can entail a negative representation of the self and negative affect (e.g., shame, humiliation) which are experienced as unbearable and psychically threatening by the subject. These affect and negative representations, when too intense to be regulated through functional emotion regulation mechanisms, can be regulated through pre-mentalistic strategies (projection or body-mediated strategies) resulting in violent behaviors aimed at avoiding psychic disorganization.

Some recent studies suggest that internalized homonegativity is influenced by romantic attachment (Jellison and McConnell, 2003; Keleher et al., 2010; Ingoglia et al., 2019; Calvo et al., 2020). People with a secure attachment have positive self-other representation, and psychic and relational resources that can promote acceptance of one’s sexual identity and protect them from internalized homonegativity (Calvo et al., 2020; Collins and Levitt, 2021). In contrast, people with insecure attachment shown negative self-other representation that can make them vulnerable to the internalization of sexual stigma. Accordingly, attachment anxiety was found to be positively associated with internalized homonegativity among both lesbian and gay people (Keleher et al., 2010; Calvo et al., 2020). As proposed by several authors (Calvo et al., 2020; Collins and Levitt, 2021), people with higher levels of attachment anxiety relies on others for personal evaluation, are sensitive to rejections, and have a negative self-representation (Santona et al., 2021) that can expose them to a self-devaluation through the internalization of social homonegative attitudes. According with the Integrated Attachment and Sexual Minority Stress Model (Cook and Calebs, 2016), it can be supposed that these internalized attitudes can further strengthen a negative representation of the self, following a vicious circle that can result in the development of an insecure attachment and negatively impact psychological wellbeing (Cook and Calebs, 2016; Collins and Levitt, 2021; Shenkman et al., 2021).

Attachment avoidance has been found to be related with increased levels of internalized sexual stigma among LG people as well (Keleher et al., 2010; Calvo et al., 2020). Although these results can be counterintuitive due to their positive self-representation, people with higher levels of attachment avoidance may show only an illusory positive self-evaluation and independence, which masks a fear of being refused and abandoned by the partner (Mikulincer and Shaver, 2005). Sexual minorities with higher levels of attachment avoidance are likely to act out distancing behaviors that, within a social context characterized by refuse and poor support, can increase their social isolation and their need of closeness, and thus undermine their defensive positive self-evaluation, exposing them to the internalization of the sexual stigma (Keleher et al., 2010).

Considering these findings, it can be supposed a complex relation between romantic attachment, internalized homonegativity and IPV perpetration among lesbian women which will be explored in the current study.

### Aims of the Study

The current study aims to explore the complex relation between romantic attachment, internalized homonegativity and SSIPV perpetration among lesbian women, in order to shed light on the mechanisms through which romantic attachment influences the risk of perpetrating couple violence.

The results have theoretical implications and can provide important information to prevention programs and services involved in the treatment of IPV perpetrators, which to date are scarce and not equipped to handle the complexities and specificities of this phenomenon among lesbian women (Rollè et al., 2021; Santoniccolo et al., 2021).

These findings can be of further importance considering that are drawn from the Italian population, where civil rights are still limited as well as inclusion and recognition of sexual minorities relationships. Despite the increasing acceptance of LGBT+ people and relationships, according with the “Annual review of the human rights situation of lesbian, gay, bisexual, trans and intersex people 2021” (ILGA Europe, 2021), Italy is ranked 35th out of the 49 European countries. For example, in Italy there are no laws to contrast homonegative attitudes, sexual discrimination, and hate speech. In addition, same-sex couples cannot get married or adopt children. The results of the current study can further shed light on characteristics and risk factors of SSIPV within the Italian population, where knowledges on these topic are still limited and the development of policies and interventions able to take care of the individual and relational wellbeing of LGBT+ people are needed.

**FIGURE 1:**
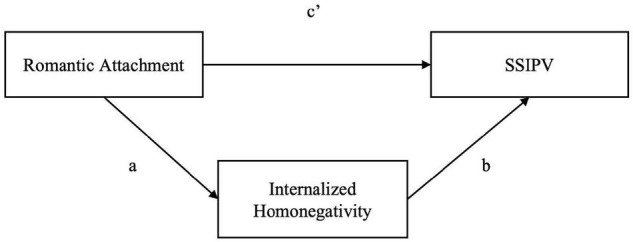
Hypothetical model. Outcome: Perpetrated Same-Sex Intimate Partner Violence (CTS2S); Mediator: Internalized Homonegativity (MISS-L); Predictor: Anxiety and Avoidance dimensions of romantic attachment (ECR-R).

According with the literature reported above (Edwards and Sylaska, 2013; Cook and Calebs, 2016; Miltz et al., 2019; Calvo et al., 2020), which suggests a complex relation between SSIPV-non-specific (i.e., romantic attachment) and SSIPV-specific risk factors (i.e., internalized homonegativity), we expect that (Figure 1):

*H1*: The relationship between romantic attachment (evaluated in both anxiety and avoidance dimensions) and SSIPV perpetration is mediated by the level of internalized homonegativity. In particular, we hypothesized that a higher level of anxiety and avoidance predicts a higher level of SSIPV perpetration and this relationship is mediated by a higher level of internalized homonegativity.
*H2*: Specifically, we expected that a higher level of internalized homonegativity positively mediated the relationship between romantic attachment and psychological aggression perpetration.
*H3*: We expected that a higher level of internalized homonegativity positively mediated the relationship between romantic attachment and physical aggression perpetration.
*H4*: We also expected that the relationship between romantic attachment and sexual coercion perpetration is positively mediated by internalized homonegativity.

## Materials and Methods

### Participants

Our sample was composed of 325 lesbian women aged 17–59 years (*M* = 29.62; SD = 7.4). The sample included 311 cisgender females (95.4%), 2 transgender women (0.6%) and 12 participants (3.7%) that indicated “Other” as gender. All the participants were involved in a romantic relationship that lasted from 1 year to 20 years.

The only two inclusion criteria we used were: (1) participants need to be involved in a lesbian relationship; (2) the romantic relationship in which they were involved lasted for at least 1 year.

Table 1 reports the sociodemographic characteristics of the sample.

**TABLE 1:** Demographic and socioeconomic characteristics of participants.

Variable	(*N* = 325) N (%)
**Age (years)**	
<20	2 (0.6%)
20–29	194 (60.1%)
30–39	91 (28.2%)
40–49	31 (9.6%)
>50	5 (1.5%)
**Duration of the relationship**	
1 year or more	86 (26.5%)
2 years or more	67 (20.6%)
3 years or more	51 (15.6%)
4 years or more	35 (10.8%)
5–10 years	64 (19.7%)
10–15 years	16 (4.9%)
15–20 years	6 (1.9%)
**Marital status**	
Unmarried	198 (60.9%)
Married	3 (0.9%)
Divorced	4 (1.2%)
Civil Union	42 (12.9%)
Cohabitation	78 (24%)
**Children**	
Yes	127 (39.1%)
No	198 (60.9%)
**Educational level**	
Secondary Education	9 (2.8%)
Short-cycle Tertiary Education	8 (2.5%)
High-school degree	125 (46.2%)
University degree	150 (46.2%)
Master/doctorate or equivalent	33 (10.2%)
**Economic status**	
Insecure	51 (15.7%)
Sufficient	176 (54.2%)
Wealthy	98 (31.1%)
**Employment status**	
Self-employed	45 (13.8%)
Employed	156 (48%)
Unemployed	23 (7.1%)
Student	101 (31.1%)

### Measures

We used the following instruments:

*Intimate Partner Violence.* We used the *Conflict Tactics Scale Short Form* (*CTS2S;* Straus and Douglas, 2004) to assess the level of violence within the couple. The CTS2S is the widely used instrument to assess intimate partner violence. The instrument is a self-report and consists of five different subscales: *Negotiation, Physical Assault, Psychological Aggression, Injury for Assault*, and *Sexual Coercion*, divided in *perpetrated* and *suffered* violence. The CTS2S has a total of 20 items and every item is rated using an 8-point Likert-type scale (*0* = *This has never happened; 1* = *Once in the past year; 2* = *Twice in the past year; 3* = *3–5 times in the past year; 4* = *6–10 times in the past year; 5* = *11–20 times in the past year; 6* = *More than 20 times in the past year; 7* = *Not in the past year, but it did happen before*). For our research purpose, we decided to use the three subscales of Psychological Aggression (PA; e.g., item*: “I insulted or swore or shouted or yelled at my partner”*), Physical Assault (PhA; e.g., item: *“I pushed, shoved, or slapped my partner”*) and Sexual Coercion [SC; e.g., item: *“I used force (like hitting, holding down, or using a weapon) to make my partner have sex”*], and the CTS2S total score (labeled as “general SSIPV”), considering only violence perpetration. The total score and the three subscales were scored using Straus and Douglas (2004) scoring method for creating frequency scores.

*Internalized Homonegativity*. We used the *Internalized Sexual Stigma for Lesbian and Gay Men* (*MISS-LG*; Lingiardi et al., 2012) to assess homonegativity in our sample. Every item is rated using a 5-point Likert-type scale, ranging from “*Totally disagree*” to “*Totally agree*.” According to Lingiardi et al. (2012), we obtained the total score by adding all the items. In our sample, the omega coefficient of the total score was 0.85, indicating a good internal consistency.

*Romantic attachment.* We used the *Experiences in Close Relationships questionnaire Revised* (ECR-R; Fraley et al., 2000; Italian version: Busonera et al., 2014) to assess the romantic attachment dimensions in our sample. The ECR-R consists of 36 items that measure two different dimensions of romantic attachment: *Avoidance of intimacy* and *Anxiety over abandonment*. The instrument uses a 7-point Likert-type scale (1 = *Strongly disagree*; 7 = *Strongly agree*), rating the extent to which each item describes how they usually feel and behave in romantic relationships. According to Busonera et al. (2014), we summed the score of items that composed the Avoidance scale and the Anxiety scale items separately. Our sample’s omega coefficient was 0.77 for the Avoidance scale and 0.61 for the Anxiety scale.

### Procedure

We used convenience sampling, and we contacted different LGBT associations to present our study to their members and ask them to participate in our research. We administered a battery of three tests to each participant, in addition to a list of questions specifically prepared to obtain general information about their sociodemographic characteristics (such as gender, age, economic status). The entire administration of the questionnaires was online through the *Qualtrics* platform during the first 6 months of 2021. At the beginning of the collection, each participant gave the authorization to use personal data and signed the written informed consent following the Italian Privacy Law n. 675/96. All the questionnaires were anonymous, and the participants were informed that they could stop participating in the study at any time. A specific mail address was created and given to participants to answer any doubts or questions. Finally, the order of the tests varied to avoid the risk of a systematic effect on the responses. All the questionnaires were digitally codified and scored based on the instructions provided by the author of each instrument.

This research did not receive any grant from funding agencies in the public, commercial, or not-for-profit sectors.

The Ethics Committee of the psychology department of Milano-Bicocca University previously approved the research project.

### Data Analysis

We conducted descriptive statistics and bivariate correlations among research variables. We performed a simple regression with the adult attachment as a predictor and the level of total SSIPV perpetration as a dependent variable and a multiple regression with adult attachment and internalized homonegativity (MISS) as predictors and SSIPV as outcome to evaluate the effect of internalized homonegativity and adult attachment on SSIPV. In particular, to address the hypothesis that the general level of homonegativity would mediate the association between attachment dimensions and SSIPV perpetration, we conducted path analyses separately for anxiety and avoidance and different types of intimate violence.

In all the path analyses the predictors were the total score of internalized homonegativity and the score of anxiety and avoidance dimensions, while the outcomes were different each time. Indeed, we performed path analyses with the total score of perpetrated SSIPV, the perpetrated psychological aggression, the perpetrated physical aggression, and the perpetrated sexual coercion as outcomes.

Multiple regression analyses were used to test whether there is a significant mediated effect, while a path analysis was subsequently conducted to visualize a general pattern of associations between all variables. Considering their association with IPV in previous studies (Rapoza and Baker, 2008; Dardis et al., 2015; Suarez et al., 2018) we also included age and duration of the relationship as confounders. Analyses were run using the statistical software IBM SPSS version 27.

## Results

### Descriptive Statistics and Path Analysis

In our sample, the 68,9% of participants perpetrated at least one act of psychological, physical, or sexual abuse during the last year. Precisely, the 67,7% of our sample reported to have perpetrated psychological SSIPV, 14.7% physical SSIPV, and 14,1% sexual SSIPV (see Table 2).

**TABLE 2:** SSIPV frequencies.

Variable	
**General SSIPV**	
At least one act of violence perpetrated	224 (68.9%)
**Perpetrated Psychological Aggression**	
Once in the past year	67 (20.7%)
Twice in the past year	81 (24.9 %)
3-5 times in the past year	50 (15.4%)
6-10 times in the past year	17 (5.2%)
11-20 times in the past year	4 (1.2%)
More than 20 times in the past year	1 (0.3%)
**Perpetrated Physical Assault**	
Once in the past year	33 (10.1%)
Twice in the past year	11 (3.4%)
3-5 times in the past year	4 (1.2%)
**Perpetrated Sexual Coercion**	
Once in the past year	28 (8.6%)
Twice in the past year	18 (5.5%)

Means, SDs and correlation values among variables are reported in Table 3.

**TABLE 3:** Descriptive statistics and bivariate correlations.

	Mean (SD)	1	2	3	4	5	6
1. Attachment Avoidance	2.62 (0.62)	–	–	–	–	–	–
2. Attachment Anxiety	3.49 (0.52)	0.307**	–	–	–	–	–
3. Internalized Homonegativity	1.39 (0.42)	0.323**	0.172**	–	–	–	–
4. General SSIPV	3.28 (3.55)	0.219**	0.127*	0.195**	–	–	–
5. Psychological SSIPV	2.63 (2.43)	0.215**	0.147**	0.206**	0.891**	–	–
6. Physical SSIPV	0.30 (1.00)	0.181**	0.019	0.131*	0.741**	0.470**	–
7. Sexual SSIPV	0.35 (1.07)	0.069	0.069	0.300	0.602**	0.249**	0.457**

*p < 0.05, **p < 0.001.

**FIGURE 2:**
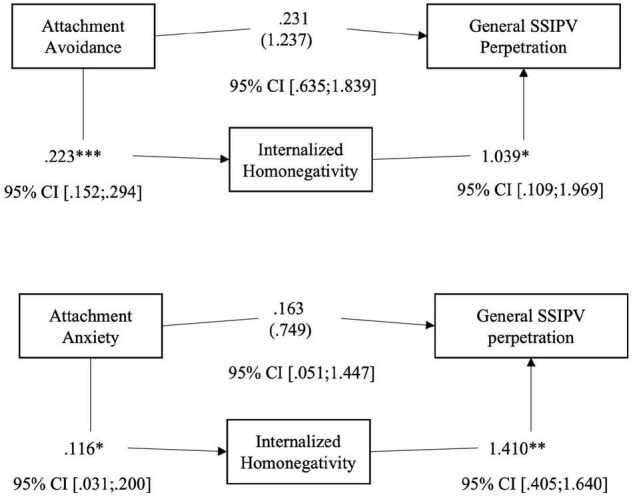
Hp.1. Path analysis with Total score of Perpetrated SSIPV as the outcome, Total score of MISS as the mediator, and the score of Anxiety and Avoidance dimensions as the predictors. *p < 0.05; **p < 0.01; ***p < 0.001.

*Hypothesis 1.* To address Hp 1, we firstly conducted a series of regression analyses to assess the possible mediation effect of internalized homonegativity in the relationship between romantic attachment and general SSIPV perpetration (see Figure 1 for hypothetical model). In our sample, the results show that both the levels of attachment’s anxiety and avoidance positively affect the level of SSIPV perpetration {*Anxiety:* [*F*(3,319) = 1.489, *p* = 0.021; *Avoidance*: *F*(3,319) = 5.451, *p* = 0.001]}. Analyzing the indirect effects, it seems that the level of anxious and avoidant attachment positively influences internalized homonegativity, which in turn positively influences general level of SSIPV perpetration (see Table 4 and Figure 2). This result means that participants with higher levels of anxiety and/or avoidance attachment seem to have more negative attitudes toward themselves as lesbians, which in turn arise the level of SSIPV perpetration within the couple.

**TABLE 4:** Defined parameters.

	General SSIPV perpetration as outcome*			Psychological SSIPV perpetration as outcome*		
	ß	p	R-square	ß	p	R-square
**Anxiety**						
Direct effect	0.586			0.507		
Total effect	0.749	0.036	0.030	0.625	0.011	0.041
**Avoidance**						
Direct effect	1.006			0.655		
Total effect	1.237	<0.001	0.051	0.843	<0.001	0.054

*Internalized homonegativity as the mediator and age and duration of relationship as cofounders.

*Hypothesis 2,3, and 4*. To better understand this association, we performed six different multivariate regression models depending on romantic attachment dimensions and IPV categories. We tested different series of regression analyses, including as predictors: (a) the *avoidance* dimension of romantic attachment (ECR-R’s Avoidance of Intimacy total score), and (b) the *anxiety* dimension (ECR-R’s Anxiety to separation total score), as mediator the general level of internalized sexual stigma (Total score of MISS-L), and as outcomes (a) the *psychological aggression* perpetration among partners (Total score of the CTS2S perpetrated psychological aggression subscale), (b) the *physical assault* perpetration among partners (Total score of the CTS2S perpetrated physical assault subscale), and (c) the *sexual coercion* perpetration among partners (Total score of the CTS2S perpetrated sexual coercion subscale).

**FIGURE 3:**
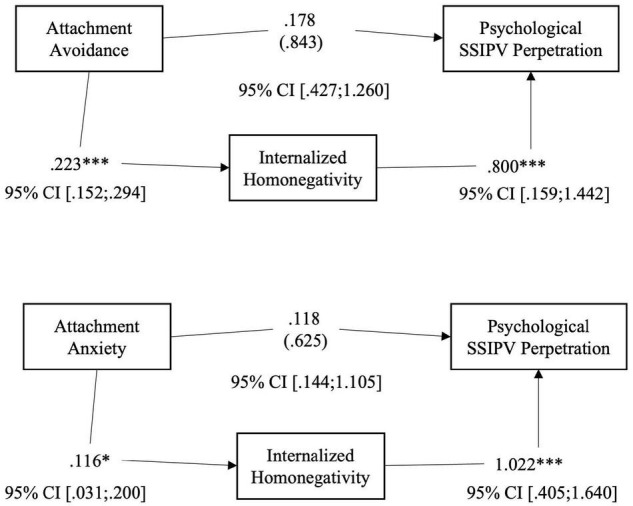
Hp. 2 Path analysis with the score of perpetrated Psychological Aggression as the outcome; the total score of MISS as the mediator, and the score of anxiety and avoidance as the predictors. *p < 0.05; ^***^p < 0.001.

**FIGURE 4:**
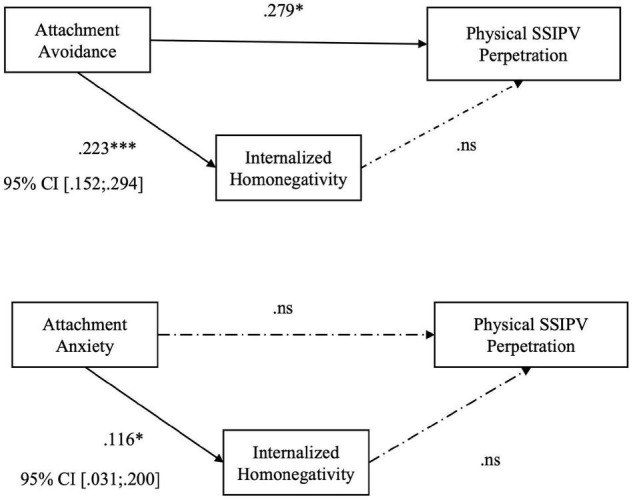
Hp. 3 Path analysis with the score of perpetrated Physical Aggression as the outcome; the total score of MISS as the mediator, and the score of anxiety and avoidance as the predictors. *p < 0.05; ***p < 0.001. ns: not significant.

**FIGURE 5:**
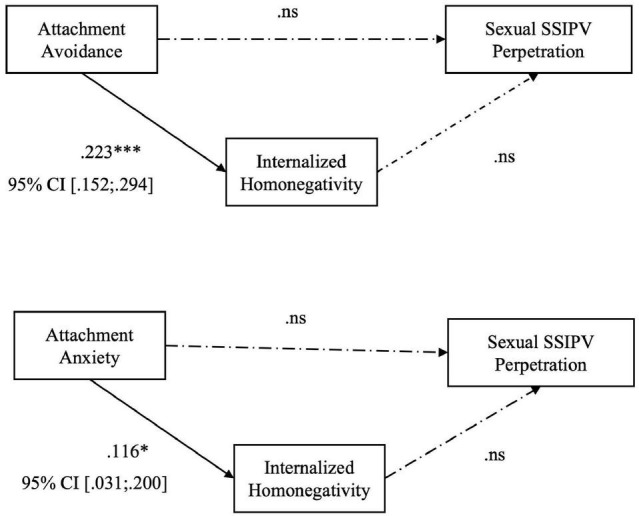
Hp. 4 Path analysis with the score of perpetrated Sexual coercion as the outcome, the total score of MISS as the mediator, and the score of anxiety and avoidance as the predictors. *p < 0.05; ***p < 0.001. ns: not significant.

Our results reveal that the level of internalized homonegativity seems to positively mediate the relationship between romantic attachment (both avoidance and anxiety) and psychological SSIPV perpetration (Hp. 2): higher levels of attachment avoidance (*B* = 0.223; *p* < 0.001), or anxiety (*B* = 0.116; p = 0.007) predicted higher levels of internalized homophobia, that in turn influences the level of psychological SSIPV perpetration within the couple (see Table 4 and Figure 3). The level of internalized homonegativity seems not to fully mediate the relationship between romantic attachment and physical assault (Hp. 3; see Figure 4) and sexual coercion (Hp. 4; Figure 5). More specifically, to test hypothesis 3, firstly we conducted two series of regressions depending on predictors (avoidance or anxiety). The first step was to test the relationship between avoidance attachment as predictor and physical assault perpetration as outcome. The result highlighted that the relationship was statistically significant: participants with higher level of attachment avoidance seemed to be more physically violent with their partners (*B* = 0.279; *p* < 0.005). Secondly, we conducted a regression using the level of avoidance dimension as predictor and the level of internalized homonegativity as outcome. Also in this case, the relationship was statistically significant: in our sample higher levels of avoidance predicts higher levels of internalized homonegativity (*B* = 0.223; *p* < 0.001). Finally, to test the mediation model, we run a regression with the avoidance dimension of attachment and the level of internalized homonegativity as predictors and the physical assault SSIPV score as outcome. The results underlined that this relationship was not statistically significant. Using the Sobel test to test the significance of the mediation model, the result suggested that the internalized homonegativity seemed not to mediate the relationship between attachment avoidance and physical assault SSIPV perpetration (Sobel test = 1.15, *p* = n.s) (see Figure 3).

We also tested the relationship between anxious attachment (as predictor) and physical SSIPV (as outcome). The results suggested that the direct association between variables was not statistically significant (see Figure 3). Then, we conducted a second regression using the level of attachment anxiety as predictor and the internalized homonegativity as outcome. Results revealed that the relationship was statistically significant: higher levels of anxiety predict higher level of internalized homonegativity (*B* = 0.116; *p* = 0.007). To test the mediation model, we used attachment anxiety and internalized homonegativity as predictors and physical SSIPV as outcome. Results underlined that the association between these variables was not statistically significant. Sobel test (test = 0.26, *p* = n.s) confirmed that the internalized homonegativity did not partially or fully mediate the association between anxiety attachment and physical SSIPV (see Figure 3).

To test Hypothesis 4 (Sexual SSIPV) we used a series of regressions. We first run a regression with the avoidance dimension of attachment as predictor and the sexual SSIPV as outcome. The results suggested that the relationship was not statistically significant. Then, we conducted a regression with attachment avoidance as predictor and internalized homonegativity as outcome. In this case the association was statistically significant: as seen before, higher level of avoidance predicts higher levels of internalized homonegativity (*B* = 0.223; *p* < 0.001). Finally, we run a regression with attachment avoidance and internalized homonegativity as predictors and sexual SSIPV as outcome. The results revealed that the relationship between variables was not statistically significant. Sobel test (test = 0.74, *p* = n.s.) confirmed that internalized homonegativity seemed not to fully or partially mediate the relationship between avoidance dimension of attachment and sexual SSIPV.

We also tested the same model using anxious attachment as predictor. The results suggested that anxious attachment did not have a direct effect on sexual SSIPV but had a positive effect on the internalized homonegativity (*B* = 0.116; *p* = 0.007). Finally, to test the mediation effect of internalized homonegativity in this relationship, we used a regression with level of attachment anxiety and internalized homonegativity as predictors and sexual SSIPV as outcome. The association between variables was not statistically significant and the Sobel test (test = 0.56, *p* = n.s.) confirmed that the level of internalized homonegativity seemed neither fully nor partially mediate the relationship between anxious attachment and sexual SSIPV.

## Discussion

The current paper explored SSIPV perpetration prevalence among lesbian women and associated factors in Italy, where only few studies have been conducted to date on this topic. Furthermore, it extends current knowledge on couple violence among lesbian women, exploring mechanisms through which romantic attachment influences SSIPV perpetration and its relationship with SSIPV-specific risk factors (i.e., internalized homonegativity).

In our sample, the 68,9% of the lesbian women perpetrated at least one act of psychological, physical, or sexual abuse. More specifically, the 67,7% reported to have perpetrated psychological SSIPV, 14,8% physical SSIPV, and 14,2% sexual SSIPV. Our results are in line with the literature on SSIPV perpetration among lesbian women (see Badenes-Ribera et al. (2016) for a meta-analysis), although methodological differences (e.g., definition of IPV, assessment tools, recall period, characteristics of the sample) can make it difficult to compare data between studies (Rollè et al., 2018). Furthermore, our prevalence rate can be underestimated due to socio-cultural reasons. Indeed, although several sexual minorities’ civil rights are now recognized in Italy, others are still not guaranteed (e.g., marriage, adoption, laws against homonegative hate behaviors), testifying the persistence of heterosexist attitudes (Calvo et al., 2020; ILGA Europe, 2021). Social homonegativity and sexual stigma which seem to persist in Italy, intertwined with the stigma related with the perpetration of SSIPV, can have limited the disclosure of participants’ actual behaviors, affecting our results.

The influence of romantic attachment on IPV among lesbian women, differed according with the form of violence considered. Specifically, attachment anxiety was directly and independently associated with general and psychological SSIPV perpetration. Similarly, attachment avoidance was directly and independently associated with general, psychological, and physical SSIPV perpetration. These results are in line with a large body of literature on both heterosexual people and sexual minorities (Bartholomew et al., 2008; Derlega et al., 2011; Gabbay and Lafontaine, 2017a,b; Velotti et al., 2018).

In contrast, attachment anxiety was not associated with physical SSIPV, and neither attachment anxiety nor attachment avoidance were associated with sexual SSIPV. The low prevalence of these forms of couple violence in our sample and the use of the short form of the CTS2 can have contributed to these results.

In addition, and more important, our study identified a partial mediation of internalized homonegativity on the association between romantic attachment and, general and psychological SSIPV perpetration. Specifically, attachment anxiety and avoidance were positively associated with internalized homonegativity, which in turn, was positively related with general and psychological SSIPV perpetration.

The results support, at least in part, the conception of violence proposed by attachment theory, demonstrating an association between romantic attachment and perpetration of couple violence. From this perspective, participants with high levels of attachment anxiety or avoidance seem to resort to violent behaviors as a dysfunctional mechanism of emotion and distance regulation to the partner (Mikulincer and Shaver, 2005; Allison et al., 2007; Bartholomew et al., 2008; Gabbay and Lafontaine, 2017a).

Furthermore, our results expand these hypotheses, highlighting how attachment anxiety and avoidance, and associated self-other representations, contribute to the internalization of the sexual stigma and thus to negative attitudes and affect toward one’s sexual identity, which in turn promote SSIPV perpetration. According with a psychodynamic conception of violence (Asen and Fonagy, 2017; Fonagy, 1999; Glasser, 1998; Yakeley, 2018), we can suppose that these unbearable negative affect toward the self, to which people with an insecure attachment are vulnerable, are regulated through pre-mentalistic strategies (resorting to violent behaviors) rather than through functional emotion regulation mechanisms. The results found by the application of the Psychological Mediation Framework (Hatzenbuehler, 2009), which demonstrated the mediational role of emotional regulation in the relation between minority stress and several dimensions of sexual minorities’ wellbeing (e.g., anxiety, depression, substance abuse) seem to further support these hypotheses.

However, it is necessary to clarify that our results were significant only in reference to general and psychological SSIPV perpetration, while they were not confirmed in relation to physical or sexual violence.

## Limitations

The current study has several limitations. First, it has a cross-sectional design which does not allow to draw firm conclusion regarding the causal direction of the associations found. Second, only self-report measures were administered in the study, and the use of the short form of the CTS can have contributed to an underestimation of the SSIPV prevalence as well as the lack of items able to detect SSIPV-related abusive tactics (e.g., threats of outing and homonegative attitudes toward the partner). Third, only lesbian women were included in the study, while other sexual minorities were not considered. In addition, considering our focus was on sexual orientation rather than on gender identity, we included also 15 participants who were not cisgender. However, no separate data were obtained on this specific group, due to the low number of participants included.

Finally, data collection was conducted during the last 6 months of 2021, when in Italy COVID-19 was still ongoing, impacting psychological and relational wellbeing, although social restriction started to lift.

## Future Directions

The current study highlights the role of romantic attachment and internalized homonegativity on SSIPV perpetrations in lesbian women. However, other studies are needed to confirm these results among other sexual identities. To make efforts to recruit representative sample can increase the generalizability of the findings emerged. Longitudinal studies are needed to confirm the causal direction here hypothesized.

Furthermore, to include other forms of violence in future studies, such as coercive control or technology-related violence, can expand our knowledge on the influence of romantic attachment and internalized homonegativity on violent behaviors.

Impact of COVID-19 pandemic and its moderating role in relation to psychological variables and SSIPV should be addressed in future studies.

In addition, future studies should assess romantic attachment through semi-structured interview in order to explore deeper representations of adult attachment bonds and their association with SSIPV. Finally, to explore the role of emotion regulation mechanisms in the association between romantic attachment, internalized homonegativity, and SSIPV perpetration, can further shed light on the results found and the theoretical considerations proposed in the current study, providing information at a clinical level.

## Conclusion

Considering the negative consequence related with SSIPV psychological victimization (e.g., Robinson, 2002; Bartholomew et al., 2008; Strickler and Drew, 2015) and its association with other forms of violence (Chong et al., 2013; Finneran and Stephenson, 2014; Wei et al., 2020), the results found provide important information at a clinical level. In order to develop interventions tailored to SSIPV perpetrators and able to reduce relapses, data highlight the need to focus on negative representations of the self, and in particular on those related with one’s sexual identity, promoting functional mechanisms of regulation of negative emotions related with the internalization of the sexual stigma. These negative representations and the associated affect, seem to flourish among people with high levels of attachment anxiety or avoidance and contribute to SSIPV perpetration as a dysfunctional mechanism of emotion regulation.

In addition, improving care providers knowledges about SSIPV, its prevalence, risk factors and specificities according with evidence-based data, can facilitate access to services for both victims and perpetrators, reducing re-victimization and further traumatic experiences. Formal sources of support are nowadays scarce and the help seeking process is still limited. This is mainly due to homonegative attitudes and a heteronormative conception of couple violence as well as by a lack of professional skills able to take care of the complexities experienced by sexual minorities victims and perpetrators of SSIPV, which seem to be further complicated by sexual stigma and minority stressors.

Accordingly, to intervene with the aim to decrease the structural stigma toward sexual minorities, to date ongoing in many societies and particularly in Italy (ILGA Europe, 2021), seems necessary in order to prevent SSIPV perpetration and promote sexual minorities’ wellbeing.

## Data Availability Statement

The data analyzed in this study is subject to the following licenses/restrictions: The dataset is still used for other papers. Requests to access these datasets should be directed to TT, tommaso.trombetta@unito.it.

## Ethics Statement

The current study involving human participants was reviewed and approved by the Ethics Committee of the Psychology Department of Milano-Bicocca University. The patients/participants provided their written informed consent to participate in this study.

## Author Contributions

GT, TT, AS, and LR: creation of the frame used in the study and research design. GT and LG: data analysis. GT, TT, LG, SR, AS, and LR: interpretation of the results. SR, AS, and LR: supervision of the entire work. All authors were involved in the discussion, writing and revision of the manuscript, and they gave the final approval of the version to be published.

## Conflict of Interest

The authors declare that the research was conducted in the absence of any commercial or financial relationships that could be construed as a potential conflict of interest.

## Publisher’s Note

All claims expressed in this article are solely those of the authors and do not necessarily represent those of their affiliated organizations, or those of the publisher, the editors and the reviewers. Any product that may be evaluated in this article, or claim that may be made by its manufacturer, is not guaranteed or endorsed by the publisher.
